# ASSESSING THE EFFECTS OF LONG-TERM CARE INSURANCE ON EQUITY AND EFFICIENCY: A CASE STUDY OF QINGDAO CITY IN CHINA

**DOI:** 10.1093/geroni/igz038.1101

**Published:** 2019-11-08

**Authors:** Wei Yang, Shuang Chang

**Affiliations:** 1 King’s College London, London, United Kingdom; 2 Tokyo Institute of Technology, Tokyo, Japan

## Abstract

The demand for equitable and efficient long-term care (LTC) has risen rapidly, and finding a suitable mechanism to finance LTC has become a pressing policy concern for many countries. A number of high income countries have chosen to use a social LTC insurance to fund the LTC system, but empirical assessments on such an insurance in low-and middle-income countries are limited. Using China as an example, this paper empirically assesses the performance of newly-piloted LTCNI by evaluating its impact on equity and efficiency in financing. We draw data from 47 in-depth interviews conducted with local government, care providers and family members of the LTCNI participants in Qingdao in 2016. We found that there remain sizable disparities in financial burden among LTCNI participants, despite of its emphasis on ensuring access to care based on people’s needs; care providers are incentivised to provide care at the least cost even this care is deemed as insufficient or inadequate due to fixed payment for their services. Our paper offers critical insights into the potential and challenges in applying LTC insurance model to a LMIC, where critical lessons can be drawn for public LTC insurance in other LMICs.

